# Caspase-Mediated Cleavage of Human Cortactin during Influenza A Virus Infection Occurs in Its Actin-Binding Domains and Is Associated with Released Virus Titres

**DOI:** 10.3390/v12010087

**Published:** 2020-01-12

**Authors:** Da-Yuan Chen, Matloob Husain

**Affiliations:** Department of Microbiology and Immunology, University of Otago, Dunedin, P.O. Box 56, Dunedin 9054, New Zealand

**Keywords:** influenza A virus, cortactin, actin-binding protein, actin-binding repeats, caspase, cathepsin

## Abstract

Influenza A virus (IAV) exploits host factors to multiply and cause disease. An in-depth knowledge of this interaction of IAV with the host will aid the development of anti-IAV intervention strategies. Previously, we demonstrated that host cortactin, an actin filament-binding protein promotes IAV infection, but undergoes degradation via a lysosome-associated apoptotic pathway during the late stages of IAV infection. Next, we wanted to further understand the mechanisms and significance of this phenomenon. By using the RNA interference screens and site-directed mutagenesis followed by western blotting, we found that lysosome protease, cathepsin C is involved in cortactin degradation in human cells infected with IAV. Furthermore, executioner apoptotic caspase, caspase-3 not caspase-6 or caspase-7 is involved in cortactin degradation during IAV infection, and caspase-3 cleavage site is located in the first actin-binding repeat of cortactin polypeptide. Finally, when expressed ectopically, the cleavage-resistant cortactin mutants decreased the amount of IAV progeny released from infected cells that was enhanced by the cleavage-sensitive cortactin wild type. These data strengthen the hypothesis proposed earlier that host cortactin plays an inhibitory role during the late stages of IAV infection, and IAV is facilitating its degradation to undermine such function.

## 1. Introduction

Influenza virus causes a significant burden and remains a threat to global public health [1,2,3]. Influenza A virus (IAV) is the most significant member of *Orthomyxoviridae* family and has been a successful human respiratory pathogen. A single-stranded, negative-sense, segmented RNA genome and a broad host range encompassing humans, birds, pigs, dogs, cats, horses, seals, and bats allow IAV to constantly circulate in nature and evolve into genetically diverse variants [4]. These variants cause regular seasonal epidemics, unpredictable pandemics, and lately frequent zoonotic outbreaks. Moreover, such evolving nature of IAV has prevented the development of a universal vaccine and aided IAV to successfully acquire the resistance against approved anti-influenza virus drugs [5,6]. The worldwide annual influenza vaccination programme, alternating in Northern and Southern Hemispheres, spearheaded by the World Health Organization (WHO) is a major tool to prevent or control seasonal influenza virus epidemics. Nevertheless, influenza virus still manages to cause significant morbidity and mortality worldwide annually [1]. In addition, recurring seasonal influenza virus epidemics cause significant loss of productivity due to work and school absenteeism and economic burden due to doctor visits and hospitalizations. 

Considering all these IAV characteristics, it will be practically impossible to eradicate IAV from the nature. Therefore, it is critical to comprehensively understand the influenza virus-host molecular interactions to develop alternative and effective anti-influenza strategies. Recently, we have identified a role of host protein named cortactin in IAV infection [7]. Cortactin is a ubiquitously-expressed protein and is expressed in most eukaryotic cells [8,9,10]. Named after its cortical intracellular distribution and binding to actin [10], cortactin is a central regulator of branched filamentous actin network [8,11], which maintains cell shape and integrity and is critical for many cellular functions such as cell motility, migration and invasion, and membrane trafficking including endocytosis. Due to its role in cell migration and invasion, cortactin is associated with various types of cancers, and overexpression of cortactin is used as a biomarker for cancer progression [7,8,11]. In addition, cortactin has also been associated with the infection of various bacterial and viral pathogens [7,8,12]. Originally identified in phosphorylated form and as a substrate of Src tyrosine kinase, cortactin is now known to be a substrate of multiple kinases, and phosphorylation plays a central role in cortactin functions [8,10]. In addition, cortactin is also known to undergo acetylation [13], which regulates its binding to filamentous actin. We have found that cortactin promotes IAV infection, but undergoes degradation by lysosome-associated caspases in infected cells [7]. This was the first such observation, and the mechanisms and significance of the involvement of cortactin during IAV infection is not entirely clear. Herein, we present the data that provide further insight into the mechanism of cortactin degradation and its significance during IAV infection.

## 2. Materials and Methods

### 2.1. Cells, Virus, and Plasmid

Madin–Darby canine kidney (MDCK) and A549 cells were grown and maintained in a complete minimum essential medium (MEM) supplemented with 10% fetal bovine serum (FBS), penicillin-streptomycin, and L-glutamine (Life Technologies) at 37 °C under a 5% CO_2_ atmosphere. Influenza virus A/New Caledonia/20/1999 (H1N1) and A/WSN/1933 (H1N1) strains were propagated in 10 day old embryonated chicken eggs and titrated on MDCK cells [7]. The plasmid expressing human cortactin-GFP fusion was a gift from Kenneth Yamada (Addgene plasmid #50728) [14], and was amplified in *E. coli* DH5α cells and purified using a plasmid purification kit (Qiagen).

### 2.2. Infection

Virus inoculum was prepared in serum-free MEM and added to cell monolayers which were prewashed twice with PBS. For infection of MDCK cells, 1 µg/mL tosylsulfonyl phenylalanyl chloromethyl ketone (TPCK)-trypsin (Sigma-Aldrich) was added to virus inoculum. After 1 h of incubation at 35 °C, virus inoculum was removed and cells were washed once with PBS. Then, fresh serum-free MEM was added and cells were incubated back at 35 °C [7].

### 2.3. Western Blotting 

Cells were lysed in a lysis buffer (50 mM Tris-HCl, pH 7.4, 150 mM NaCl, 0.5% SDS, 0.5% sodium deoxycholate, 1% Triton X-100, and 1x protease inhibitor cocktail [Roche]). The amount of protein was quantitated using a BCA kit (Thermo) [7]. Then, equal amounts of protein were resolved on 10% Tris-glycine SDS-PAGE and transferred onto Protran^®^ Premium nitrocellulose membrane (GE healthcare). The membrane was then probed with mouse anti-cortactin (1:500; #05-180, clone 4F11, Millipore), rabbit anti-cortactin (1:500; #3502, Cell Signaling), rabbit anti-GFP (1:1000; #632592, Takara), rabbit anti-β-actin (1:2000; #ab8227, Abcam) or goat anti-NP (1:10,000; G150, kindly provided by Richard Webby, St Jude Children’s Research Hospital, Memphis (TN), USA) antibody followed by horseradish peroxidase-conjugated anti-mouse IgG (1:5000; #626520, Life Technologies), anti-rabbit IgG (1:5000; #A16023, Life Technologies) or IRDye 680RD-conjugated anti-goat IgG (1:50,000; #926-32214, LI-COR) antibody. The protein bands that were visualized by chemiluminescence or fluorescence and images were acquired on Odyssey Fc imaging system (LI-COR). Images were then exported as TIFF files, minimally adjusted for brightness and contrast, and composed in figures in Adobe Photoshop CC 2015 [7,15].

### 2.4. Quantitative Real-Time RT-PCR 

Total RNA from the cells was isolated by using Nucleospin RNA isolation Kit (Macherey-Nagel) and the cDNA was synthesised using PrimeScript RT reagent kit (Takara) by following the manufactures’ protocol. Quantitative real-time PCR was performed on ViiA 6 Real-Time PCR system (Applied Biosystems) using SYBR Green Select Master Mix (Life Technologies) and predesigned KiCqStart primers for mentioned caspases and cathepsins (Sigma-Aldrich). The primers for human beta-actin were 5′-GACGACATGGAGAAAATCTG-3′ (Forward) and 5′-ATGATCTGGGTCATCTTCTC-3′ (Reverse) [15].

### 2.5. RNA Interference 

Predesigned small-interfering RNA (siRNA) oligonucleotides and a nontargeting MISSION control siRNA (Sigma-Aldrich) were used to deplete the expression of mentioned human caspases and cathepsins. The siRNA oligonucleotides were delivered to cells by reverse transfection using Lipofectamine RNAiMAX (Life Technologies) by following the manufactures’ suggested protocol. Briefly, siRNA oligonucleotides (10 nM) and Lipofectamine RNAiMAX (2 µL) were diluted separately in an OptiMEM I medium (Life Technologies), mixed together, and incubated for at least 20 min at room temperature. The siRNA-RNAiMAX complex was then mixed with cell suspension which was then transferred to a cell culture plate. Cells were incubated at 37 °C for 72 h before further processing or infection.

### 2.6. Site-Directed Mutagenesis

The Asp → Glu substitution in each putative caspase cleavage motif was done by a single point mutation (GAT → GAA or GAC → GAG) using the site-directed ligase-independent mutagenesis (SLIM) approach described elsewhere [16]. Briefly, four primers (two long, containing the desired mutation and two short) were designed for creating each mutation. A PCR reaction was then performed using these primers, GFP-cortactin plasmid template and AccuPrime Pfx DNA polymerase (Life Technologies). The PCR product was then subjected to a series of reactions: DpnI (New England Biolabs) digestion, denaturation, and hybridization. Finally, the hybridized PCR product was transformed to *E. coli* DH5α cells, colonies were screened, and point mutations were confirmed by DNA sequencing. The correct plasmids were then further amplified, purified, and used for ectopic expression followed by western blotting screening. 

### 2.7. Ectopic Expression 

The plasmids were delivered to cells by reverse transfection using Lipofectamine 2000 (Life Technologies) by following the manufacturers’ suggested protocol. Briefly, plasmid DNA (2 µg) and Lipofectamine 2000 (3 µL) were diluted separately in an OptiMEM I medium (Life Technologies), mixed together, and incubated for at least 15 min at room temperature. The plasmid DNA-Lipofectamine 2000 complex was then mixed with cell suspension which was then transferred to a cell culture plate. Cells were then incubated at 37 °C for 48 h before further processing or infection. 

### 2.8. Microplaque Assay 

Culture medium from the infected cells was collected and centrifuged at 17,000× *g* for 1 min at room temperature to remove the cell debris. The cleared culture medium supernatant was then mixed with 0.3% bovine serum albumin (Sigma-Aldrich). Ten-fold serial dilutions of culture medium was prepared and each dilution was used as inoculum to infect the confluent monolayers of MDCK cells in a 48-well culture plate (Corning) for 1 h at 35 °C. After removing the viral inoculum, cells were overlaid with serum-free MEM supplemented with 1 µg/mL TPCK-trypsin and 0.8% Avicel (RC-581, FMC Biopolymer), and incubated back at 35 °C for 18 to 24 h. The overlay was removed from the cells and cells were fixed with 4% formalin (Sigma-Aldrich) for 20 min and permeabilized with 0.5% Triton X-100 for 5 min at room temperature. Cells were then stained with mouse anti-NP antibody (1:1000; #MAB8251, Millipore) followed by horseradish peroxidase-conjugated anti-mouse IgG antibody (1:1000; #626520, Life Technologies). Finally, plaques were visualized by adding the TrueBlue peroxidase substrate (KPL). 

### 2.9. Statistical Analysis

All statistical analyses were performed using GraphPad Prism (version 6 or 8). The *p*-values were calculated using unpaired t-tests for pairwise data comparisons and one-way analysis of variance (ANOVA) for multiple data set comparisons. A *p*-value of ≤ 0.05 was considered significant.

## 3. Results

### 3.1. Lysosomal Protease, Cathepsin C is Involved in Cortactin Degradation during IAV Infection 

In our previous report [7], we utilised lysosome and caspase inhibitors to identify the role of lysosome-associated caspases in cortactin degradation in IAV-infected cells. Lysosome has long been described to be involved in inducing the apoptosis under various conditions [17,18], and lysosomal proteases such as cathepsin B have been shown to contribute to both intrinsic and extrinsic apoptotic pathways [19,20,21]. To further understand the role of this pathway, we sought to identify the cathepsins involved in cortactin degradation during IAV infection. For this, we performed a RNA interference screen and depleted the expression of representative cathepsins: Cathepsin B, C, D, G, H, K, O, and W [22], in human lung alveolar cells A549 using validated predesigned small-interfering RNA (siRNA) obtained from Sigma-Aldrich. The A549 cells depleted with the expression of individual cathepsins were then infected with influenza virus A/WSN/1933/(H1N1) strain (hereafter referred as WSN), and the level of cortactin polypeptide was compared in uninfected and infected cells by western blotting (WB) using actin as loading control. This initial screen indicated the involvement of cathepsin C in cortactin degradation during IAV infection. Out of the cathepsins that were targeted, the depletion of cathepsin C expression rescued the level of cortactin polypeptide in infected cells when compared to uninfected cells (Figure S1). 

**Figure 1:**
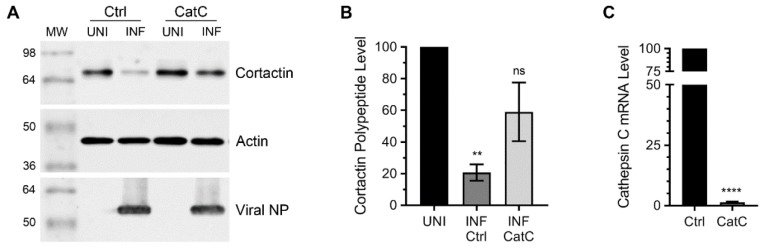
The depletion of cathepsin C rescued cortactin polypeptide degradation in IAV-infected cells. (**A**) A549 cells were transfected with 10 nM of control (Ctrl) small-interfering RNA (siRNA) or cathepsin C (CatC) siRNA for 72 h. Cells were then infected with WSN at a multiplicity of infection (MOI) of 3.0. After 24 h, total cell lysates of uninfected (UNI) and infected (INF) cells were prepared, and cortactin, actin, and viral nucleoprotein (NP) polypeptides were detected by western blotting (WB). (**B**) The cortactin and actin bands were quantified using Image Studio Lite software (LI-COR) and the levels of cortactin were normalized with the corresponding actin levels. Then, the normalized level of cortactin in the respective uninfected sample was considered 100% to determine its level in the corresponding infected sample. (**C**) A549 cells, transfected with control or cathepsin C siRNA, were processed to detect cathepsin C and actin mRNA levels by qPCR. Then, the levels of cathepsin C mRNA in control siRNA transfected sample and cathepsin C siRNA transfected sample were normalized with corresponding actin mRNA levels. Finally, the normalized level of cathepsin C mRNA in the control siRNA transfected sample was considered 100% to determine the cathepsin C mRNA level in cathepsin C siRNA transfected sample. The error bars represent the means ± standard errors of the means of three biological replicates. The asterisks represent *p*-values stated in the text and calculated by ANOVA (**B**) or unpaired t-test (**C**) and indicate significant differences in the means. MW: Molecular weight; ns: Nonsignificant.

To quantify such recovery of cortactin polypeptide, we repeated the WB experiment with cathepsin C-depleted cells (Figure 1A), and the intensity of cortactin and actin polypeptide bands was quantified using Image Studio Lite software (Version 5.0, LI-COR). Then, the amount of cortactin was normalized with the corresponding actin amount. Finally, the normalized amount of cortactin polypeptide in uninfected cells was considered 100% to determine its amount in infected cells. We found that compared to a significant 79% (*p* = 0.004) reduction in cortactin polypeptide level in infected cells transfected with the control siRNA, there was only a nonsignificant 41% reduction in cortactin polypeptide level in infected cells transfected with the cathepsin C siRNA (Figure 1B). In other words, the level of cortactin polypeptide in infected cells recovered from 21% to 59% after the depletion of cathepsin C expression, which was confirmed by quantitative real-time PCR (qPCR) (Figure 1C).

### 3.2. Caspase-3, not Caspase-6 or Caspase-7 is Involved in Cortactin Degradation during IAV Infection

**Figure 2:**
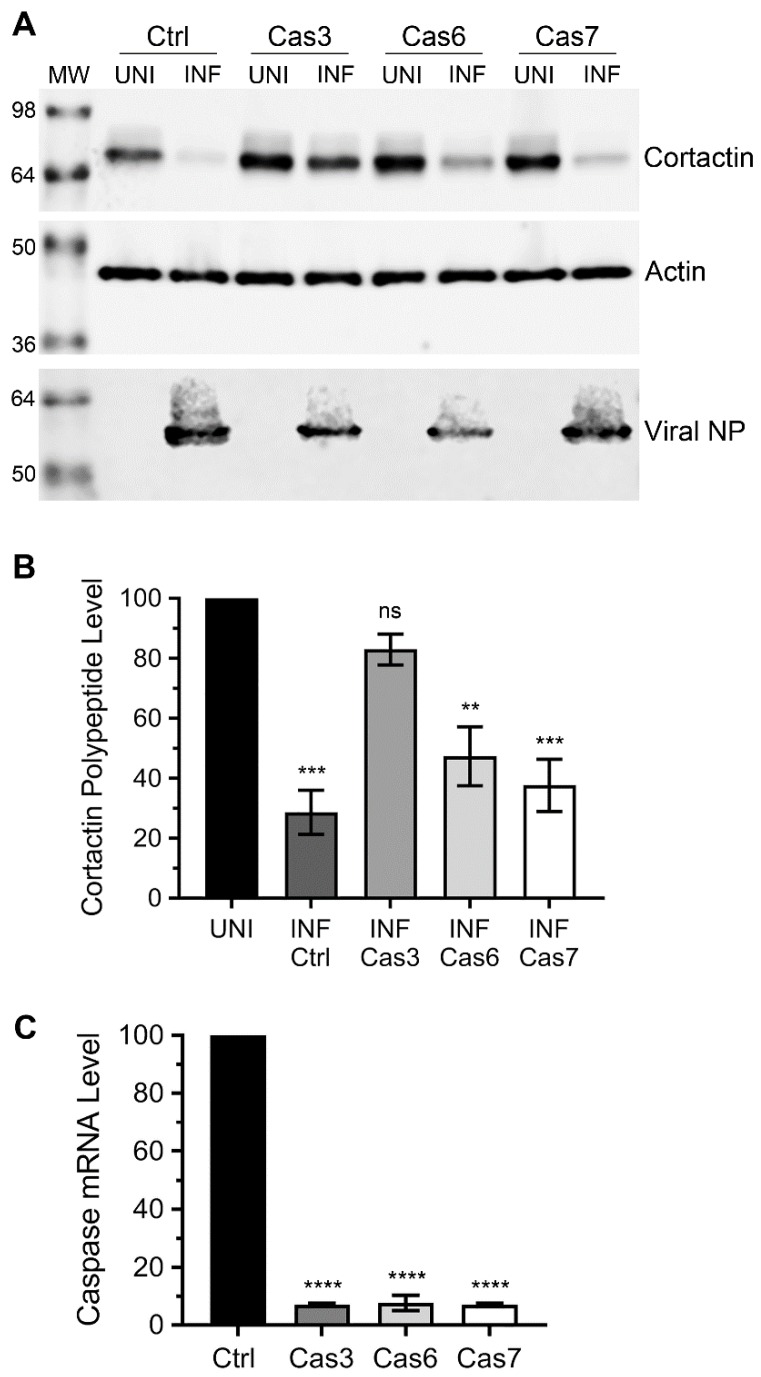
The depletion of caspase-3 rescued cortactin polypeptide degradation in IAV-infected cells. (**A**) A549 cells were transfected with 10 nM of control (Ctrl), caspase-3 (Cas3), caspase-6 (Cas6), or caspase-7 (Cas7) siRNA for 72 h. Cells were then infected with WSN at a multiplicity of infection (MOI) of 3.0. After 24 h, total cell lysates of uninfected (UNI) and infected (INF) cells were prepared, and cortactin, actin, and viral NP polypeptides were detected by WB. (**B**) The cortactin and actin bands were quantified and normalized with actin as described in Figure 1B. Then, the normalized level of cortactin in the respective uninfected sample was considered 100% to determine its level in the corresponding infected sample. (**C**) The caspase-3, -6, or -7 mRNA levels in A549 cells transfected with control or caspase-3, -6 or -7 siRNA, were detected and normalized with actin mRNA level as described in Figure 1C. Then, the normalized level of caspase-3, -6, or -7 mRNA in control siRNA transfected cells was considered 100% to determine their levels in caspase-3, -6, or -7 siRNA transfected cells, respectively. Error bar represents the means ± standard errors of the means of three biological replicates. The asterisks represent *p*-values mentioned in the text calculated by ANOVA and indicate the significant differences in means; ns: Nonsignificant.

As mentioned above, we previously used a caspase-3 inhibitor to identify the involvement of caspase-3 in cortactin degradation during IAV infection [7]. However, such an inhibitor could target other caspases too. To ascertain the involvement of caspase-3 and assess whether other executioner caspases, caspase-6 or -7 were involved in cortactin polypeptide degradation in response to IAV infection, we employed RNA interference and depleted the expression of caspase-3, -6, or -7 [23,24] in A549 cells using predesigned siRNA obtained from Sigma-Aldrich. Then, the cells were infected with WSN, and the level of cortactin polypeptide was analysed and quantified by WB as described above. Consistent with previous data [7], the depletion of caspase-3, but not caspase-6 or caspase-7 expression significantly rescued the level of cortactin polypeptide in infected cells (Figure 2A). Specifically, compared to a significant 71% (*p* = 0.0001) reduction in cortactin polypeptide level in infected cells transfected with the control siRNA, there was only a nonsignificant 17% reduction in cortactin polypeptide level in infected cells transfected with the caspase-3 siRNA (Figure 2B). In other words, the level of cortactin polypeptide in infected cells recovered from 29% to 83% after the depletion of caspase-3 expression. Whereas, there was no significant recovery in the cortactin polypeptide level in infected cells depleted with caspase-6 or caspase-7 expression (Figure 2B). The depletion of caspase-3, -6, -7 expression was confirmed by qPCR (Figure 2C).

### 3.3. Caspase Cleavage Sites are Located within Actin-Binding Repeat 1 of Cortactin Polypeptide

**Figure 3:**
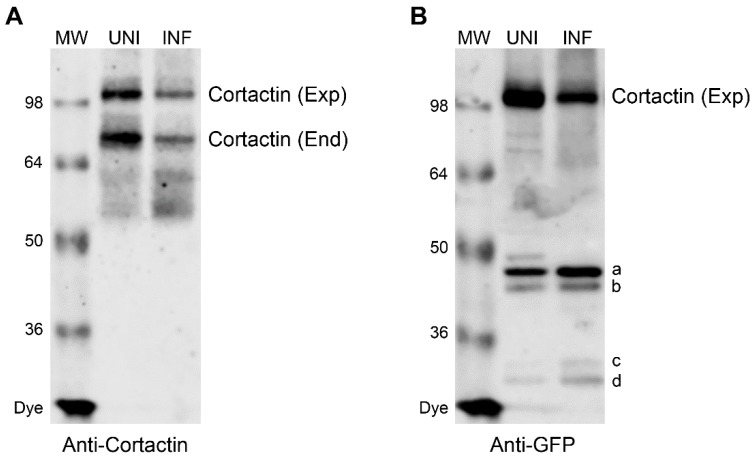
WB profile of the ectopically expressed GFP-cortactin polypeptide. MDCK cells were transfected with plasmid expressing the GFP-cortactin fusion for 48 h and subsequently infected with WSN at a MOI of 3.0. After 24 h, total cell lysates of uninfected (UNI) and infected (INF) cells were prepared, and cortactin polypeptide was detected by WB using either anti-cortactin antibody (**A**) or anti-GFP antibody (**B**). Exp: ectopically expressed cortactin; End: endogenously expressed cortactin. Letters a, b, c, and d indicate cleaved cortactin fragments.

We next endeavoured to identify the location of caspase cleavage sites in cortactin polypeptide using site-directed mutagenesis. Earlier, we could not detect a cleavage product of endogenous cortactin polypeptide in IAV infected cells by WB using the anti-cortactin antibody [7] (Figure 3A). We envisaged that cortactin cleavage occurs sequentially and at multiple locations resulting into fragments which are too small to detect under the WB conditions employed. This notion was supported by the identification of 34 putative caspase cleavage motifs in human cortactin polypeptide by CaspDB database [25] (Table 1). Furthermore, four smaller fragments, marked with letters a, b, c, and d (Figure 3B), were observed when cortactin polypeptide fused with green fluorescent protein (GFP) *N*-terminally was ectopically expressed from a plasmid in WSN-infected cells, and detected by WB using the anti-GFP antibody. (These fragments were also observed at a lower level in uninfected cells, presumably because of a basal level of caspase activation due to plasmid transfection). 

This indicated that cortactin polypeptide is getting cleaved at caspase cleavage sites that are located at the *N*-terminus, and the resulting cleavage products became sufficiently larger and detectable by WB after the addition of GFP (27 kDa). Therefore, to identify the caspase cleavage sites, we used this WB profile of wild type (WT) GFP-cortactin polypeptide as a template to screen ectopically expressed cortactin mutants containing Asp (D) → Glu (E) mutation in P1 position of each putative caspase cleavage motif, XXXD (Table 1, Figure S2). Out of 33 such cortactin mutants screened, we identified two mutations, D93E and D116E (Figure S2, indicated in bold) in actin-binding repeat 1 [26] that rescued the full-length GFP-cortactin polypeptide from degradation in WSN-infected cells. Furthermore, the D93E mutation resulted in the disappearance of fragment b, whereas the D116E mutation shifted the mobility of the fragment a upwards in WB profile (Figure S2, indicated by asterisks).

**Figure 4:**
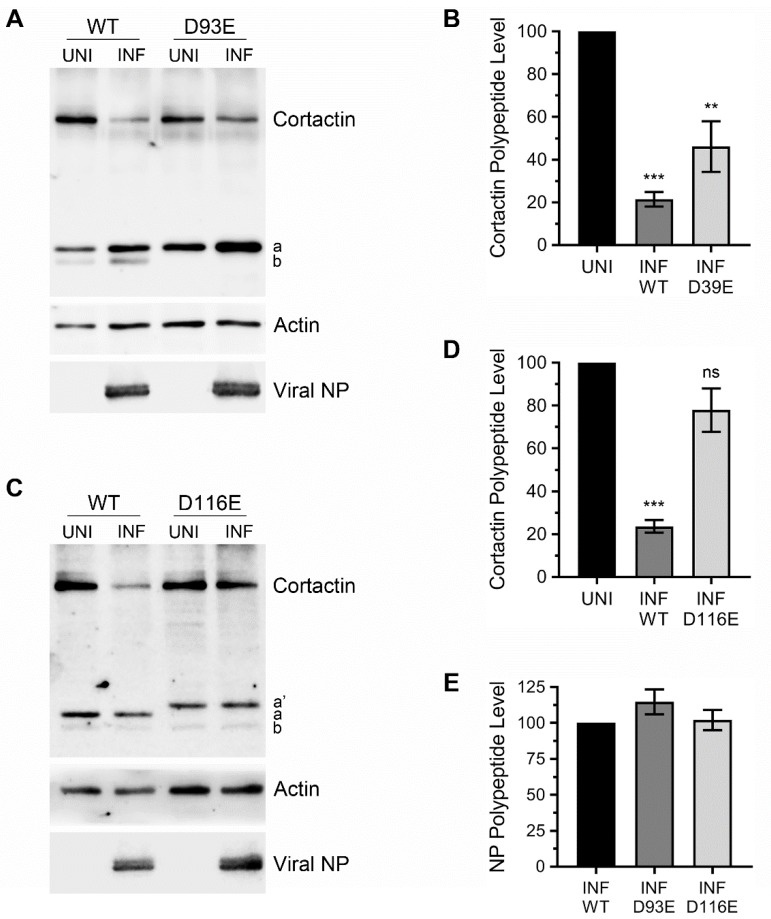
The D93E and D116E mutations rescued cortactin polypeptide degradation in IAV infected cells. MDCK cells were transfected with plasmid expressing the wild type (WT) GFP-cortactin or GFP-cortactin containing D93E (**A**) or D116E (**C**) mutations for 48 h and subsequently infected with WSN at a MOI of 3.0. After 24 h, total cell lysates of uninfected (UNI) and infected (INF) cells were prepared, and cortactin (using anti-GFP antibody), actin, and viral NP polypeptides were detected by WB. (**B**,**D**,**E**) The cortactin, NP and actin bands were quantified and normalized with actin as described in Figure 1B. Then, the normalized level of WT cortactin and D93E cortactin (**B**) or D116E cortactin (**D**) in the respective uninfected sample was considered 100% to determine their levels in the corresponding infected sample. (**E**) The normalized level of NP in WT cortactin infected sample was considered 100% to determine its level in D93E cortactin or D116E cortactin infected sample. Error bar represents the means ± standard errors of the means of three biological replicates. The asterisks represent *p*-values mentioned in the text calculated by ANOVA and indicate the significant differences in means; ns: Nonsignificant. Letters a and b indicate cleaved cortactin fragments; letter a’ indicates mobility shift in fragment a.

Next, we quantified the recovery in the full-length cortactin polypeptide level from degradation as a consequence of D93E and D116E mutations. For this, we overexpressed these two mutants alongside the WT GFP-cortactin and infected the cells with WSN, and GFP-cortactin polypeptide levels were analysed and quantified by WB as above. Both mutations though D93E (Figure 4A) less efficiently than D116E (Figure 4C), rescued the cortactin polypeptide from degradation during IAV infection (Figure 4). Consistent with reduction in endogenous cortactin polypeptide level above, the level of ectopically expressed GFP-cortactin WT polypeptide was reduced by a significant 79% (*p* = 0.0004) in response to IAV infection (Figure 4B). However, the level of GFP-cortactin D93E polypeptide was reduced by only 54% (though still significant, *p* = 0.003) in response to IAV infection (Figure 4B). Likewise, compared to a significant 76% (*p* = 0.0002) reduction in GFP-cortactin WT polypeptide level, the level of GFP-cortactin D116E polypeptide was reduced by only a nonsignificant 22% in response to IAV infection (Figure 4D). In other words, D93E and D116E mutations recovered the GFP-cortactin polypeptide levels in infected cells from 21% to 46% and from 24% to 78%, respectively. There was no significant difference in viral NP polypeptide levels between WT cortactin expressing cells and D93E or D116E mutant cortactin-expressing cells (Figure 4E)), indicating that overexpression of these mutants do not affect viral protein expression. 

### 3.4. Ectopically Expressed Cortactin D93E and D116E Mutants Decrease the Amount of IAV Progeny Released from Infected Cells that is Enhanced by Cortactin WT 

**Figure 5:**
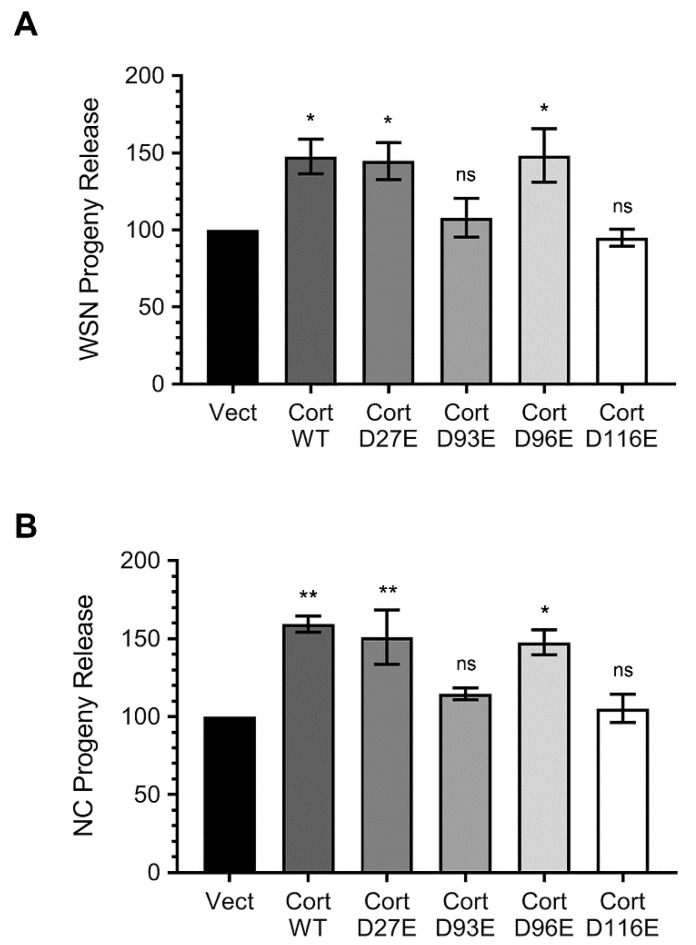
The degradation-resistant cortactin mutants, D93E and D116E decrease the amount of IAV progeny released from infected cells. (**A**,**B**) MDCK cells were transfected with empty plasmid pEGFP-C1 (Vect), plasmid expressing wild type (WT) GFP-cortactin or GFP-cortactin containing D27E, D93E, D96E or D116E mutations for 48 h. Cells were then infected with WSN (**A**) or NC (**B**) at an MOI of 3.0. After 24 h, the culture media from each cell were harvested and the amount of WSN (**A**) or NC (**B**) progeny released was measured by the microplaque assay. Then, the amount of progeny released from empty plasmid pEGFP-C1 transfected cells was considered 100% to compare the amount of progeny released from the cells transfected with indicated cortactin plasmid. Error bar represents the means ± standard errors of the means of three biological replicates. The asterisks represent *p*-values mentioned in the text calculated by ANOVA and indicate the significant differences in means; ns: Nonsignificant.

Previously, by using the RNA interference-mediated depletion and ectopic expression of cortactin, we demonstrated that host cortactin promotes IAV infection [7]. We also hypothesised that due to its branched filamentous actin-stabilising function that could be deleterious to IAV progeny assembly and release, cortactin is degraded during the late stages of IAV infection [7]. If this hypothesis was to be true, then the two degradation-resistant cortactin mutants, D93E and D116E generated here would interfere with the titres of IAV progeny released from infected cells. To test this, we expressed these two mutants alongside empty vector, degradation-sensitive WT cortactin and other degradation-sensitive cortactin mutants, D27E and D96E, and subsequently infected the cells with either WSN or influenza virus A/New Caledonia/20/1999/(H1N1) (hereafter referred to as NC) strains. After 24 h, the viral progeny released from these cells into the culture medium was quantified by microplaque assay. Consistent with previous observations, compared to empty vector expressing cells, there was a significant 48% (*p* = 0.04) and 45% (*p* = 0.02) and 48% (*p* = 0.0002) increase in the amount of WSN progeny released from the cells expressing degradation-sensitive WT cortactin and D27E and D96E mutants, respectively (Figure 5A). However, compared to empty vector expressing cells, there was no significant change in the amount of WSN progeny released from the cells expressing degradation-resistant D93E and D116E mutants (Figure 5A). Similarly, compared to empty vector expressing cells, there was a significant 59% (*p* = 0.002) and 51% (*p* = 0.007) and 48% (*p* = 0.01) increase in the amount of NC progeny released from the cells expressing WT cortactin and D27E and D96E mutants, respectively, and no significant change from the cells expressing D93E and D116E mutants (Figure 5B). These data, at least partly support our above-stated hypothesis. However, further investigations are needed to understand the actin filament-virion assembly/release dynamics on the surface of the cells expressing degradation-sensitive and degradation-resistant cortactin. 

## 4. Discussion

Following on our previous findings [7], we have provided herein a further insight into the mechanism and significance of cortactin degradation during IAV infection. Using the RNA interference screen, we have confirmed the involvement of lysosome and identified the role of a lysosomal protease, cathepsin C in cortactin degradation during IAV infection. However, the role of other cathepsins (A, E, F, L, S, V, and X [18,22]) not targeted here cannot be ruled out. Furthermore, using the same strategy, we also confirmed the involvement of mainly caspase-3 not caspase-6 or -7, the other two main executioner caspases [24], in cortactin degradation. Next, it will be interesting to investigate the link between cathepsin C and caspase-3 in the degradation of cortactin during IAV infection. Cathepsin C, also known as dipeptidyl peptidase I, is one of the first lysosomal proteases to be discovered and is ubiquitously expressed, including in the lungs. It is the main regulator of multiple pro-inflammatory serine proteases, activation of which leads to tissue damage and inflammatory response [27]. One of the cathepsin C-regulated serine proteases, proteinase-3 has been described to cleave and activate caspase-3 [28]. Hence, there is a link between lysosome and apoptosis or cathepsin C and caspase-3 activation, however such link has to be established during IAV infection. Further, several viral proteins contribute to controlling the apoptosis in IAV infected cells [29]; it will be interesting to identify the viral protein(s) which is controlling the apoptotic pathway that is leading to cortactin degradation. 

We also endeavoured to identify the caspase cleavage motif(s) on cortactin polypeptide using site-directed mutagenesis. The cortactin polypeptide has well-defined multifunctional domains: *N*-terminal acidic domain followed by 6.5 tandem actin-binding repeats (each 37 aa long), helical domain, proline-rich region, and *C*-terminal SH3 domain [11]. Cortactin binds to actin branching promoter complex, Arp2/3, and filamentous actin through its *N*-terminal acidic domain and actin-binding repeats, respectively, and promotes and stabilises the branched filamentous actin network [30,31]. The actin-binding repeats of cortactin also possess the acetylation sites [13]. Through its *C*-terminal SH3 domain, cortactin interacts with proteins such as cortactin-binding protein 90 (CBP-90), zona occludens-1 (ZO-1), neural Wiskott-Aldrich syndrome protein (N-WASP), and Dynamin 2 that are involved in signal transduction, cytoskeleton regulation, and membrane trafficking [11]. Further, cortactin undergoes phosphorylation on several sites spanning the helical domain and proline-rich region [11]. The phosphorylation and acetylation conversely regulate the cortactin folding and actin-binding functions [13,32]. Apparently, each of these cortactin domains has a putative caspase cleavage motif (Table 1). Particularly, each of the cortactin actin-binding repeats possesses at least one caspase cleavage motif (Table 1). This suggests the complete removal of actin-binding and consequently branched filamentous actin-stabilising capability of cortactin during the late stages of IAV infection. Indeed, consistent with this hypothesis, the two cortactin mutants, D93E and D116E out of 33 screened that rescued the cortactin degradation in response to IAV infection were located in actin-binding repeat 1 [26]. In other words, the cleavage of cortactin polypeptide occurs at caspase motifs VEQD (position 90–93) and SQVD (position 113–116) located within actin-binding repeat 1. However, we believe that cleavage on these motifs occurs independently of each other, because the fragment a did not disappear from WB profile of D93E mutant (Figure 4A) and the fragment b did not disappear from D116E WB profile (Figure 4C). Further, the cleavage of cortactin polypeptide is potentially not limited to only these two motifs, because we did not detect a larger cortactin polypeptide fragment. Our working model is that the first major cleavage in cortactin polypeptide occurs at SQVD motif (position 113–116) that exposes the rest of the polypeptide for further cleavage at VQMD motif (position 127–130) and DRVD motif (position 130–133) and onwards (Table 1) by caspase-3 or other caspases. This model finds support from the data obtained with cortactin double mutants D116/130E (Figure S2) and D116/133E (not shown). While no change was observed in the WB profile of cortactin single mutants D130E or D133E, the mobility of fragment a shifted further upwards when these mutations were created along with D116E mutation (Figure S2, asterisk). Nonetheless, several additional simultaneous mutations in multiple caspase cleavage motifs of cortactin polypeptide are needed to propose a complete model of cortactin degradation during IAV infection. 

Finally, we found that the degradation-resistant cortactin D93E and D116E mutants interfered with the amount of IAV progeny released from infected cells that was enhanced by the cleavage-sensitive cortactin WT. These data further supported our hypothesis that cortactin likely has a dual, but contrasting role during IAV infection. Cortactin, in association with Arp2/3 complex and N-WASP, promotes and stabilises the branching of filamentous actin [30], which regulate both clathrin-dependent and clathrin-independent endocytosis [11]. The IAV is known to use receptor-mediated endocytosis to enter the host cell [33]. Hence, we believe that in the early stages of infection intact cortactin is required for IAV entry to maintain dynamic branched filamentous actin network for efficient endocytosis of incoming virions [7]. However, during the late stages of IAV infection intact cortactin is potentially deleterious due to the same branched filamentous actin-associated function. The late stages of IAV infection, assembly, budding, and release of viral progeny from the plasma membrane microdomains (lipid rafts) potentially require a less dynamic and unbranched filamentous actin network. This argument finds support in earlier observations where filamentous actin was found to be re-organised close to the IAV assembly sites [34]. Further, such re-organised filamentous actin promotes the clustering of viral proteins close to the IAV assembly sites and was important for efficient IAV assembly and budding [34]. Furthermore, the disruption of filamentous actin dynamism by drugs that either inhibit actin polymerization or enhance actin depolymerization led to an increase in the amount of IAV progeny released from infected cells [34]. Potentially, the degradation-resistant cortactin mutants interfered with such filamentous actin reorganization and/or dynamism and consequently in the IAV progeny assembly and release. Nevertheless, further molecular and microscopic investigations are needed to elucidate such dual role of cortactin during IAV infection. 

In conclusion, the data presented here has strengthened our argument that host cortactin is degraded during IAV infection by a lysosome-associated apoptotic pathway. The initial cleavage of cortactin polypeptide occurs at the *N*-terminus in actin-binding repeat 1, and cleavage-resistant cortactin interferes with the IAV progeny release from infected cells. Further elucidation of the precise role of cortactin in IAV infection may reveal potential antiviral targets.

## Figures and Tables

**Table 1:** Mutagenesis of CaspDB-predicted caspase cleavage sites in human cortactin polypeptide.

No.	Domain	P1	P5–P5′	Score	P5–P5′ (Post Mutation)	Remark
1	NTA	D15	SIAQ**D**-DAGAD	0.795	SIAQ**E**-DAGAD	
2		D20	DAGA**D**-DWETD	0.510	DAGA**E**-DWETD	
3		D27	ETDP**D**-FVNDV	0.898	ETDP**E**-FVNDV	
4	Actin-Binding Repeats	D93	GVEQ**D**-RMDKS	0.851	GVEQ**E**-RMDKS	Full-length recovery
5		D96	QDRM**D**-KSAVG	0.927	QDRM**E**-KSAVG	
6		D116	CSQV**D**-SVRGF	0.948	CSQV**E**-SVRGF	Full-length recovery
7		D130	GVQM**D**-RVDQS	0.828	GVQM**E**-RVDQS	
8		D133	MDRV**D**-QSAVG	0.851	MDRV**E**-QSAVG	
9		D153	ASQKD-YSSGF	0.881	ASQK**E**-YSSGF	
10		D167	GVQA**D**-RVDKS	0.775	GVQA**E**-RVDKS	Failed to generate
11		D170	ADRV**D**-KSAVG	0.904	ADRV**E**-KSAVG	
12		D177	AVGF**D**-YQGKT	0.916	AVGF**E**-YQGKT	
13		D190	ESQRD-YSKGF	0.890	ESQR**E**-YSKGF	
14		D207	KDKV**D**-KSAVG	0.925	KDKV**E**-KSAVG	
15		D227	ESQK**D**-YVKGF	0.800	ESQK**E**-YVKGF	
16		D241	GVQT**D**-RQDKC	0.788	GVQT**E**-RQDKC	
17		D244	TDRQ**D**-KCALG	0.642	TDRQ**E**-KCALG	
18		D264	ESQK**D**-YKTGF	0.794	ESQK**E**-YKTGF	
19		D281	SERQ**D**-SAAVG	0.963	SERQ**E**-SAAVG	
20		D288	AVGF**D**-YKEKL	0.914	AVGF**E**-YKEKL	
21		D301	ESQQ**D**-YSKGF	0.955	ESQQ**E**-YSKGF	
22		D315	GVQK**D**-RMDKN	0.536	GVQK**E**-RMDKN	
23		D318	KDRM**D**-KNAST	0.884	KDRM**E**-KNAST	
24	Helical	D326	STFE**D**-VTQVS	0.636	STFE**E**-VTQVS	
25		D365	KEQE**D**-RRKAE	0.595	KEQE**E**-RRKAE	
26	Proline-Rich	D423	PVYE**D**-AASFK	0.792	PVYE**E**-AASFK	
27		D452	MEAA**D**-YREAS	0.751	MEAA**E**-YREAS	
28		D483	YPAE**D**-STYDE	0.711	YPAE**E**-STYDE	
29		D487	DSTY**D**-EYEND	0.767	DSTY**E**-EYEND	
30		D492	EYEN**D**-LGITA	0.581	EYEN**E**-LGITA	Full-length mobility shift ↓
31	SH3	D502	VALY**D**-YQAAG	0.901	VALY**E**-YQAAG	
32		D508	QAAG**D**-DEISF	0.578	QAAG**E**-DEISF	
33		D516	SFDP**D**-DIITN	0.572	SFDP**E**-DIITN	Full-length mobility shift ↑
34		D526	IEMI**D**-DGWWR	0.829	IEMI**E**-DGWWR	

P1 position indicates the cleavage sites in caspase cleavage motifs. P5–P5′ indicates the amino acid sequence preceding and following P1 position. The cleavage motifs are underlined and cleavage sites are in bold. NTA: *N*-terminal acidic; SH3: Src homology 3.

## Supplementary Materials

The following are available online at https://www.mdpi.com/1999-4915/12/1/87/s1, Figure S1: RNA interference screening to identify the cathepsin(s) involved in cortactin degradation in IAV infected cells, Figure S2: WB screening to identify the cortactin mutants resistant to degradation in IAV infected cells.
