# Supplementary material for: Caspase-Mediated Cleavage of Human Cortactin during Influenza A Virus Infection Occurs in Its Actin-Binding Domains and Is Associated with Released Virus Titres
Source: Viruses. 2020 Jan 12;12(1):87. doi: 10.3390/v12010087 (PMC7019683; doi:10.3390/v12010087)
Supplement: Supplementary file 1 [file viruses-12-00087-s001.pdf]

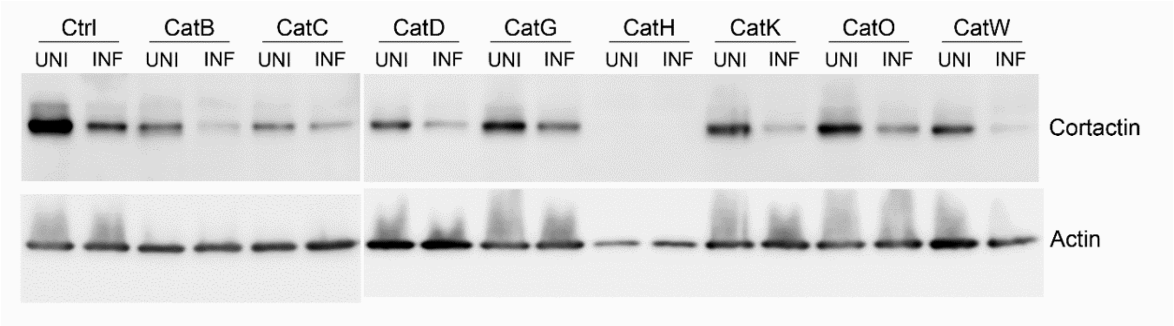

Figure S1. RNA interference screening to identify the cathepsin(s) involved in cortactin degradation in IAV infected cells. A549 cells were transfected with 10 nM of control (Ctrl) siRNA or siRNAs targeting human cathepsin (Cat) B, C, D, G, H, K, O, or W for 72 h. Cells were then infected with WSN at a multiplicity of infection (MOI) of 3.0. After 24 h, total cell lysates of uninfected (UNI) and infected (INF) cells were prepared, and cortactin and actin polypeptides were detected by WB.

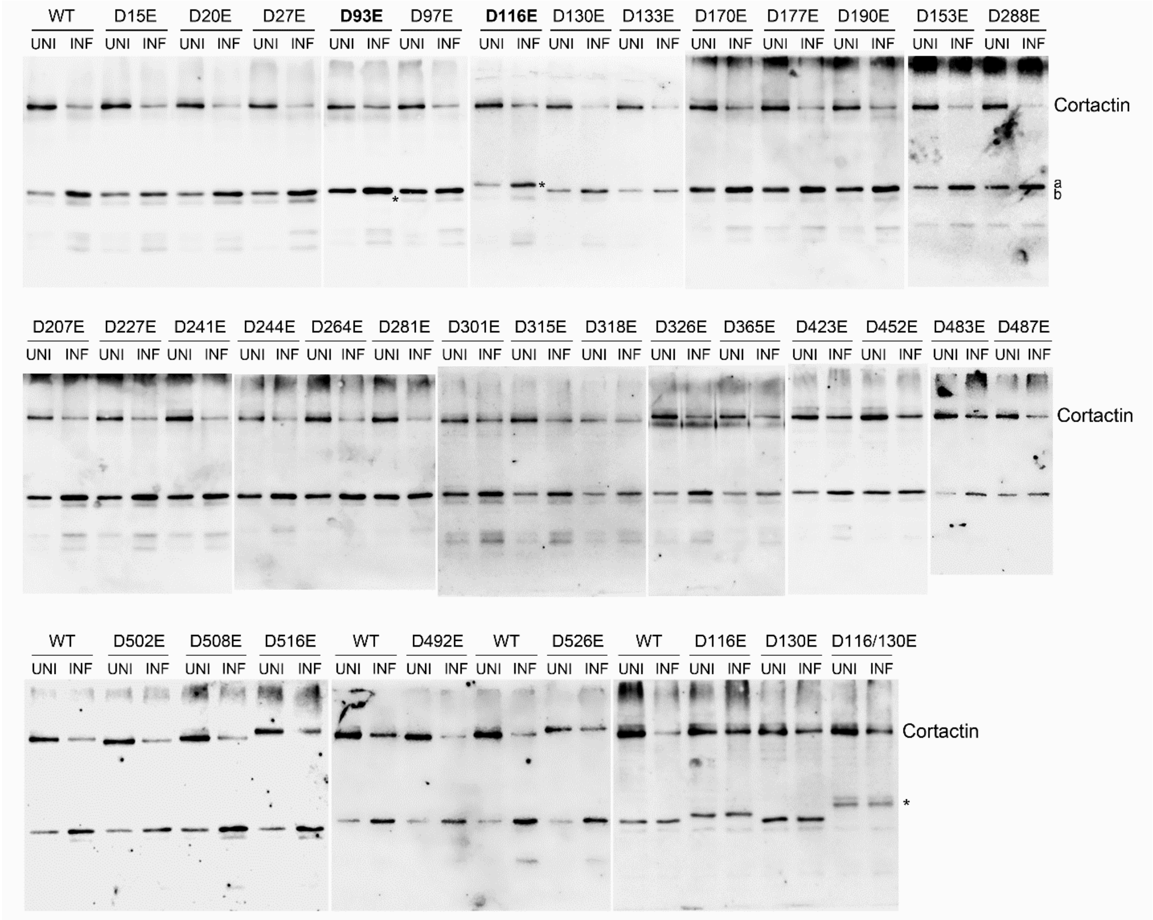

Figure S2. WB screening to identify the cortactin mutants resistant to degradation in IAV infected cells. MDCK cells were transfected with plasmid expressing the WT GFP-cortactin or GFP-cortactin containing indicated mutation for 48 h and subsequently infected with WSN at a MOI of 3.0. After 24 h, total cell lysates of uninfected (UNI) and infected (INF) cells were prepared, and cortactin polypeptide was detected by WB using anti-GFP antibody. Bold letters indicate a hit; asterisks indicate change in cleaved cortactin fragments. Each blot contained the WT GFP-cortactin UNI/INF lanes as control; however, these lanes have been spliced from some blots to accommodate them in the figure.
